# *Toxoplasma gondii* Requires Glycogen Phosphorylase for Balancing Amylopectin Storage and for Efficient Production of Brain Cysts

**DOI:** 10.1128/mBio.01289-17

**Published:** 2017-08-29

**Authors:** Tatsuki Sugi, Vincent Tu, Yanfen Ma, Tadakimi Tomita, Louis M. Weiss

**Affiliations:** a Department of Pathology, Albert Einstein College of Medicine, Bronx, New York, USA; b Department of Medicine, Albert Einstein College of Medicine, Bronx, New York, USA; University of Pittsburgh

**Keywords:** amylopectin, bradyzoite, latency, metabolism, *Toxoplasma gondii*, energy utilization, glycogen metabolism, glycogen phosphorylase, posttranslational modification, protein phosphorylation

## Abstract

In immunocompromised hosts, latent infection with *Toxoplasma gondii* can reactivate from tissue cysts, leading to encephalitis. A characteristic of *T. gondii* bradyzoites in tissue cysts is the presence of amylopectin granules. The regulatory mechanisms and role of amylopectin accumulation in this organism are not fully understood. The *T. gondii* genome encodes a putative glycogen phosphorylase (TgGP), and mutants were constructed to manipulate the activity of TgGP and to evaluate the function of TgGP in amylopectin storage. Both a stop codon mutant (Pru/TgGP^S25stop^ [expressing a Ser-to-stop codon change at position 25 in TgGP]) and a phosphorylation null mutant (Pru/TgGP^S25A^ [expressing a Ser-to-Ala change at position 25 in TgGp]) mutated at Ser25 displayed amylopectin accumulation, while the phosphorylation-mimetic mutant (Pru/TgGP^S25E^ [expressing a Ser-to-Glu change at position 25 in TgGp]) had minimal amylopectin accumulation under both tachyzoite and bradyzoite growth conditions. The expression of active TgGP^S25S^ or TgGP^S25E^ restored amylopectin catabolism in Pru/TgGP^S25A^. To understand the relation between GP and calcium-dependent protein kinase 2 (CDPK2), which was recently reported to regulate amylopectin consumption, we knocked out CDPK2 in these mutants. Pru*Δcdpk2*/TgGP^S25E^ had minimal amylopectin accumulation, whereas the *Δcdpk2* phenotype in the other GP mutants and parental lines displayed amylopectin accumulation. Both the inactive S25A and hyperactive S25E mutant produced brain cysts in infected mice, but the numbers of cysts produced were significantly less than the number produced by the S25S wild-type GP parasite. Complementation that restored amylopectin regulation restored brain cyst production to the control levels seen in infected mice. These data suggest that *T. gondii* requires tight regulation of amylopectin expression for efficient production of cysts and persistent infections and that GP phosphorylation is a regulatory mechanism involved in amylopectin storage and utilization.

## IMPORTANCE

*Toxoplasma gondii* is an obligate intracellular parasite that causes disease in immune-suppressed individuals, as well as a fetopathy in pregnant women who acquire infection for the first time during pregnancy. This parasite can differentiate between tachyzoites (seen in acute infection) and bradyzoites (seen in latent infection), and this differentiation is associated with disease relapse. A characteristic of bradyzoites is that they contain cytoplasmic amylopectin granules. The regulatory mechanisms and the roles of amylopectin granules during latent infection remain to be elucidated. We have identified a role of *T. gondii* glycogen phosphorylase (TgGP) in the regulation of starch digestion and a role of posttranslational modification of TgGP, i.e., phosphorylation of Ser25, in the regulation of amylopectin digestion. By manipulating TgGP activity in the parasite with genome editing, we found that the digestion and storage of amylopectin due to TgGP activity are both important for latency in the brain.

## INTRODUCTION

*Toxoplasma gondii* is the causative agent of toxoplasmosis. Uncontrolled replication of this parasite as tachyzoites during infection, either reactivation or acute, causes encephalitis or pneumonia in immunocompromised patients and can cause death or defects in central nervous system (CNS) development during congenital infection (1). Although immunocompetent humans can eventually suppress tachyzoite replication, conversion of this parasite to bradyzoites (within tissue cysts) results in chronic infection, and these latent organisms evade control by the host immune system (1). Current drug treatment cannot eradicate bradyzoites. The prevalence of the latent infection of this parasite can be as high as 70% in various human populations (2). Latent infection in animals used for meat serves as a major source for human infection (3). These widespread latent parasites become problematic when patients become immunocompromised. Understanding how the parasites facilitate persistent infection should provide important new insights into control strategies for this life cycle stage.

**FIG 1:**
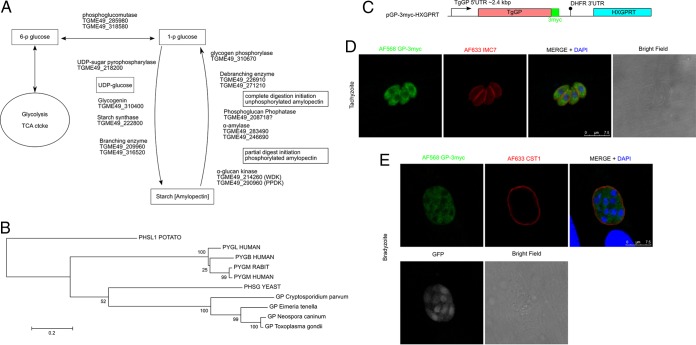
TgGP is expressed in parasite cytosol. (A) TgGP in the amylopectin metabolism pathway. Enzyme identifiers were retrieved from the KEGG database. (B) Phylogenetic analysis of alpha-glucan phosphorylases (UniProt ID, short name) from human (P11217, PYGM; P11216, PYGB; and P06737, PYGL), rabbit (P00489, PYGM), potato (P04045, PHSL1), yeast (P06738, PHSG), *Neospora caninum* (F0VMV1, NcGP), *Toxoplasma gondii* (S8EYF4, TgGP), *Eimeria tenella* (U6KXX5, EtGP), and *Cryptosporidium parvum* (Q5CX54, CpGP). (C) Schema of TgGP-3myc-expressing plasmid. The TgGP genomic locus, including the 5′ UTR for the native promoter, was fused with a C-terminal 3myc tag. The plasmid contains the hypoxanthine-xanthine-guanine phosphoribosyl transferase (HXGPRT) selectable marker cassette. DHFR, dihydrofolate reductase. (D and E) Parasites expressing TgGP-3myc were inoculated to HFF host cells and incubated for 24 h under normal culture conditions for tachyzoites (D) or incubated for 2 h to allow the parasites to invade host cells, followed by incubation under conditions of pH 8.2 medium without CO_2_ for 4 days for bradyzoites (E). Cells were stained with anti-MYC rabbit antibody (detected by Alexa 568-conjugated secondary antibody, shown in green pseudocolor) and anti-IMC7 mouse polyclonal antibody (D) or anti-CST1 mouse monoclonal antibody (E) (detected by Alexa 633-conjugated secondary antibody, shown in red pseudocolor). DAPI was used to visualize nuclei (blue). For the bradyzoite conditions, the bradyzoite stage-specific LDH2 promoter-driven GFP signal was detected to verify the parasites were bradyzoites. GFP signal is shown in grey scale, not merged. Scale bar, 7.5 μm.

One of the ultrastructural characteristics of bradyzoites is that they have polysaccharide granules in their cytosol (4). The polysaccharide granules contain sugar polymers in the form of amylopectin (5, 6). Amylopectin is also found in several other *T. gondii* life stages, including macrogametes, oocysts, and sporozoites (4). The function of amylopectin storage in this organism is not known. In *Eimeria tenella* and *Cryptosporidium parvum*, a relation between the amylopectin content of the parasite and the infectivity of sporozoites has been reported (7, 8). Examination of a curated database of metabolic pathways, LAMP ([Liverpool] Library of Apicomplexan Metabolic Pathways) (9), suggests that a complete amylopectin metabolism pathway is encoded in *T. gondii* (Fig. 1A).

To regulate the amount of starch, the regulation of glycogen phosphorylase (GP) (EC 2.4.1.1) activity is a conserved strategy in eukaryotic cells (10, 11). GP, which in other eukaryotes is enzymatically involved in starch consumption, is predicted to be encoded by TGME49_310670. In mammalian cells, GP activity is altered by allosteric regulation with the cofactor of AMP, activated by the phosphorylation of Ser14 at the amino terminus, and inhibited by glucose (12). Yeast GP is similarly regulated by Thr29 phosphorylation at the amino terminus (11). *Toxoplasma* GP has been reported to be phosphorylated in a published phosphoproteomic survey (13). *Toxoplasma gondii* protein kinases, such as *T. gondii* cyclic AMP (cAMP)-dependent protein kinase catalytic subunit 3 (TgPKAc3), have been demonstrated to be involved in tachyzoite/bradyzoite differentiation (14). *T. gondii* calcium-dependent protein kinase 2 (TgCDPK2) has been reported to be involved in amylopectin regulation (15). It is not clear how *T. gondii* regulates the enzymes involved in amylopectin metabolism to establish a regulated system of amylopectin storage and digestion.

In the present report, we evaluate how *T. gondii* utilizes a phosphorylation signal to regulate the activity of its GP (TgGP) and what would occur if this parasite lacked amylopectin digestion and storage. To answer these questions, we introduced point mutations at a phosphorylation site in TgGP that we predicted would be involved in the regulation of TgGP enzymatic activity to create mutants with inactive or hyperactive GP, tested these mutant parasites in an *in vivo* mouse model infection to evaluate the effects of manipulation of TgGP on amylopectin digestion/storage, and elucidated the effects of amylopectin abnormalities on the ability of this parasite to develop a latent infection in the murine brain.

## RESULTS

### *Toxoplasma gondii* glycogen phosphorylase is involved in amylopectin digestion.

To investigate the role of amylopectin regulation in this parasite, we focused on TgGP, which is a digestive enzyme for starch (amylopectin/amylose/glycogen) (Fig. 1A) (9). Sequence analysis demonstrated that *Toxoplasma* has a single conserved GP ortholog (TGME49_310670 [TgGP]). This putative GP ortholog is conserved in the apicomplexans *Neospora*, *Eimeria*, *Hammondia*, *Cyclospora* and *Cryptosporidium* but is not found in *Plasmodium*, *Babesia*, or *Theileria* (http://www.EuPathdb.org). Phylogenetic analysis demonstrates that the GP enzymes from the apicomplexan parasites form a distinct node that is different from mammalian, yeast, and plant GPs (Fig. 1B).

To characterize the localization of TgGP, C-terminally 3myc-tagged TgGP was expressed from the native promoter of TgGP (Fig. 1C). In the Pru*Δ;ku80Δ;hxgprt* strain (tachyzoite conditions), parasites expressed the TgGP-3myc in their cytosol in a patchy manner (Fig. 1D), consistent with a previous report by Uboldi et al. of a tagged form of TgGP in the RH strain (15). In the bradyzoite stage, parasites also expressed GP-3myc in the cytosol (Fig. 1E).

**FIG 2:**
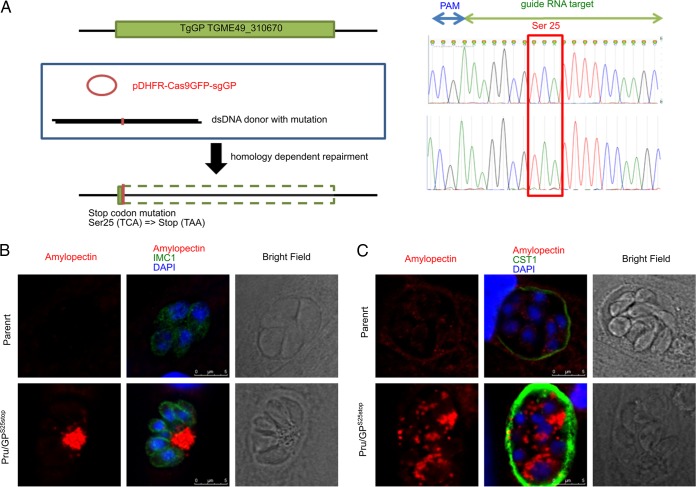
Replacement of Ser25 of TgGP by site-directed point mutation. (A) (Left) Schematic diagram of the CRISPR/Cas9-driven site-directed point mutation at the TgGP native locus. The Cas9 construct expressing the sgRNA targeting TgGP and a donor sequence with a mutation were cotransfected, and transfected parasites with the DHFR drug-selectable marker on the Cas9 construct were selected by using pyrimethamine. (Right) Sequence chromatogram of the mutated locus. The sgRNA target sequence and protospacer adjacent motif (PAM) sequence in the complement strand are shown by blue and green arrows. The codon locus for wild-type Ser25 is marked by the red box. (B and C) Amylopectin accumulation in the parental parasites and GP knockout parasites was detected with PAS staining. Parasites were inoculated to HFF host cells and incubated for 24 h under normal culture conditions for tachyzoites (B) or incubated for 2 h to allow the parasites to invade host cells, followed by incubation under conditions of pH 8.2 medium without CO_2_ for 4 days for bradyzoites (C). Cells were stained with PAS, followed by immunofluorescence assay (IFA) with anti-IMC1 rabbit polyclonal antibody (B) or anti-CST1 mouse monoclonal antibody (C). DAPI was used to visualize nuclei (blue). After the PAS-staining procedure, the GFP signal is bleached. Scale bars show 5 μm.

To identify the function of this enzyme in amylopectin digestion, we introduced a stop codon point mutation at the N-terminal Ser25 position of TgGP in the native genome locus (Fig. 2A and Table 1) of the Pru*Δ;ku80Δ;hxgprt T. gondii* strain by using clustered regularly interspaced short palindromic repeats–CRISPR-associated protein 9 (CRISPR-Cas9). Point mutations were successfully introduced into parasites to make mutants with a stop codon (Pru/GP^S25stop^) (Fig. 2A). This stop codon mutation of Ser25→stop (TCA→TAA) at the N-terminal position in TgGP resulted in the parasites accumulating amylopectin under tachyzoite culture conditions, whereas the parental parasites did not accumulate any amylopectin, as demonstrated by periodic acid-Schiff (PAS) staining (Fig. 2B). This amylopectin accumulation seen in GP knockout parasites was further enhanced by bradyzoite culture conditions, and amylopectin filled the parasite cell bodies (Fig. 2C), whereas parental parasites had the normal distribution of amylopectin, with some PAS-positive vacuoles in the cytosol (Fig. 2C). These data suggest that TgGP is an active enzyme that digests amylopectin in tachyzoites, and the knockout phenotype resulting from the stop codon mutation at Ser25 is consistent with data on a TgGP knockout reported by Uboldi et al. (15).

**TABLE 1:** Transgenic parasites in this study

Strain	Description^a^	Parental strain
Parent	Pru*Δku80Δhxgprt*	
Pru/GP1^S25stop^	TGME49_310670@73TCA→TAA	Parent
Pru/GP1^S25S^	TGME49_310670@73TCA→TCC	Parent
Pru/GP1^S25A^	TGME49_310670@73TCA→GCA	Parent
Pru/GP1^S25E^	TGME49_310670@73TCA→GAA	Parent
Pru/GP1^S25A^::GP1^S25A^-3myc	GP1^S25A^-3myc is expressed	Pru/GP1^S25A^
Pru/GP1^S25A^::GP1^S25S^-3myc	GP1^S25S^-3myc is expressed (complemented strain)	Pru/GP1^S25A^
Pru/GP1^S25A^::GP1^S25E^-3myc	GP1^S25E^-3myc is expressed	Pru/GP1^S25A^
PruΔ*cdpk2*	TGME49_225490@46CCA→TGA	Parent
Pru*Δcdpk2*/GP1^S25S^	TGME49_310670@73TCA→TCC	Pru/GP1^S25S^
	TGME49_225490@46CCA→TGA	
Pru*Δcdpk2*/GP1^S25A^	TGME49_310670@73TCA→GCA	Pru/GP1^S25A^
	TGME49_225490@46CCA→TGA	
PruΔ*cdpk2*/GP1^S25E^	TGME49_310670@73TCA→GAA	Pru/GP1^S25E^
	TGME49_225490@46CCA→TGA	

a @, for the mutated locus, the triplet codon and the base number from the start codon are shown with the resultant mutated codon. ToxoDB identifiers: TGME49_310670, TgGP; TGME49_225490, TgCDPK2.

### Phosphorylation of Ser25 in TgGP is crucial for amylopectin digestion.

**FIG 3:**
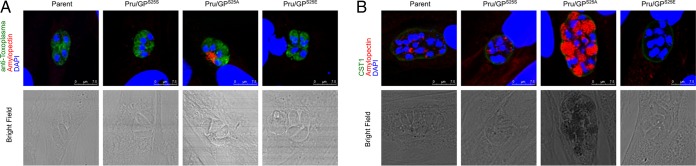
TgGP Ser25 is critical for regulation of amylopectin digestion. TgGP mutants were inoculated to HFF host cells and incubated for 24 h under normal culture conditions for tachyzoites (A) or incubated for 2 h to allow the parasites to invade host cells, followed by incubation under conditions of pH 8.2 medium without CO_2_ for 4 days for bradyzoites (B). Parasites were stained with PAS (red), followed by immunofluorescence detection of parasite proteins with anti-*Toxoplasma* antibody (A) or anti-CST1 monoclonal antibody (B) (green). Nuclei were stained with DAPI (blue). Scale bars show 7.5 µm.

Eukaryotic GPs are known to be regulated by a posttranslational modification, N-terminal phosphorylation (12). Phosphoproteome data indicated that the N terminus of TgGP has an N-terminally phosphorylated Ser25 (13). Sequence alignments suggested that the N-terminal sequences of TgGP and other apicomplexan GPs around this phosphorylation site are well conserved and similar to the N-terminal sequences of mammalian GPs (see Fig. S1 in the supplemental material). Thus, we focused on this phosphorylation site as a potential regulatory site for the amylopectin digestion activity of TgGP. To dissect the roles of amylopectin digestion and storage in the parasite, we produced a parasite mutant with an inactive TgGP, which resulted in the accumulation of amylopectin in the parasite, and a mutant with a hyperactive TgGP, which resulted in a lack of amylopectin storage. We introduced a site-directed point mutation at Ser25 of TgGP in the native genome locus of the parental parasite line Pru*Δku80Δhxgprt* as described in Fig. 1A, using different donor sequences in order to produce various amino acid substitutions. Ser25 was replaced with Ala (GP^S25A^) to make that site nonpermissive for phosphorylation or with Glu (GP^S25E^) to mimic the phosphorylated-Ser state. The parental strain, as well as a silent mutant that maintained S25S, did not show any amylopectin accumulation under the tachyzoite culture conditions. The S25A mutant displayed amylopectin accumulation in residual bodies and in its cytosol (Fig. 3A). The S25E phosphorylation-mimetic mutant did not have any amylopectin accumulation under tachyzoite culture conditions (Fig. 3A). When the parasites were incubated under bradyzoite induction culture conditions (pH 8.2 and CO_2_ depletion without NaHCO_3_ in the culture medium), parasites with the wild-type TgGP peptide sequence (parent and Pru/GP^S25S^ strains) had amylopectin accumulation in their cytosol (Fig. 3B), the S25A mutant (Pru/GP^S25A^) had amylopectin accumulation which filled the majority of its cytosolic space (Fig. 3B), and the phosphorylation-mimetic parasite (Pru/GP^S25E^) had no amylopectin accumulation (Fig. 3B). Quantitative analysis of amylopectin using PAS staining confirmed an increase (*P* < 0.001) in the Pru/GP^S25A^ mutant and a decrease (*P* < 0.05) in the Pru/GP^S25E^ mutant compared to the amylopectin levels in parasites containing the wild-type TgGP (parent and Pru/GP^S25S^ strains) peptide sequence (Fig. S2A). All of the mutants, when cultured under bradyzoite induction conditions, displayed a CST1-positive cyst wall (Fig. 3B), demonstrating that all of the mutants could undergo bradyzoite differentiation in response to bradyzoite culture conditions. The bradyzoite conversion rate, as measured by the number of CST1-positive vacuoles, was not significantly changed in any of the mutants (Fig. S3), which further illustrates that bradyzoite differentiation itself was not altered by the mutations introduced at the TgGP locus. Collectively, these results from mutant parasites indicate that GP^S25A^, which could not be phosphorylated, had reduced GP activity and GP^S25E^, which mimicked phosphorylated Ser25, had hyperactive GP.

10.1128/mBio.01289-17.1 FIG S1  GP N-terminal regions are not conserved throughout organisms. The following glycogen phosphorylase sequences were retrieved from Uniprot (http://www.uniprot.org) with the indicated protein identifiers. Human liver-type GP (HUMAN_PYGL: P06737), muscle GP (HUMAN_PYGM: P11217), and brain GP (HUMAN_PYGB: P11216), rabbit muscle GP (RABBIT_PYGM: P00489), yeast GP (YEAST_PHSG: P06738), potato GP (POTATO_PHSL1: P04045), *Neospora caninum* GP (NcGP: F0VMV1), *Toxoplasma gondii* GP (TgGP: S8EYF4), *Eimeria tenella* GP (EtGP: U6KXX5), and *Cryptosporidium parvum* GP (CpGP: Q5CX54). Sequences were aligned with ClustalW and colored according to the characteristics of the amino acids (glycine: yellow, proline: brown, negative charge: red, positive charge: blue, bulky: green, polar uncharged: grey). Conserved sites are shown in shaded characters. The N-terminal phosphorylation site for the GP activity regulation (Ser14 in the mammalian GP) is marked with a pound sign (#). Two positively charged amino acid sites for the interaction with phosphorylated N-terminal Ser in the mammalian GP (R43 and R69) are marked with an asterisk (*). (Please note that mammalian GP amino acid numbering takes into consideration the removal of the starting Met.) N-terminal regions corresponding to positions 1 to 176 of mammalian GPs are shown in the figure. Download FIG S1, TIF file, 2.8 MB. Copyright © 2017 Sugi et al. 2017 Sugi et al. This content is distributed under the terms of the Creative Commons Attribution 4.0 International license.

10.1128/mBio.01289-17.2 FIG S2  Quantitation of amylopectin staining in various *Toxoplasma gondii* mutants. (A) *Toxoplasma gondii* GP mutants (Kruskal-Wallis chi-square value = 140.27, df = 3, *P* < 2.2e−16). (B) *Toxoplasma gondii* ΔCDPK GP mutants (Kruskal-Wallis chi-square value = 558.06, df = 3, *P* < 2.2e−16). Amylopectin was quantified by taking the mean fluorescence intensity of the PAS staining seen in cysts that developed *in vitro* under bradyzoite culture conditions (see Materials and Methods). Cysts were identified by the presence of CST1 (3) staining. The number (*n*) under each box plot indicates the number of cysts that were quantified. Asterisks indicate significant differences between strains: *P* ≤ 0.001; ***, *P* ≤ 0.05; NS, no significant difference. Download FIG S2, TIF file, 1.6 MB. Copyright © 2017 Sugi et al. 2017 Sugi et al. This content is distributed under the terms of the Creative Commons Attribution 4.0 International license.

10.1128/mBio.01289-17.3 FIG S3  Bradyzoite conversion rate of GP mutants. Parental Pru*Δ;ku80Δ;hxgprt* and Pru/GP^S25S^, Pru/GP^S25A^ and Pru/GP^S25E^ were inoculated to HFF host cell monolayers and incubated for 2 h, followed by culture under the indicated culture conditions of pH 7.4 (normal culture condition for tachyzoite culture) or pH 8.2 (bradyzoite induction condition; see the detailed induction conditions in Materials and Methods) for 48 h. After incubation, cells were fixed and CST1-positive cyst walls were stained with anti-CST1 antibody and then counterstained with anti-*Toxoplasma* rabbit antibody. At least 100 parasitophorous vacuoles per sample were counted to calculate the CST1-positive-vacuole rate. Means and standard deviations from three independent experiments are shown. Statistical analysis using one-way ANOVA did not detect significant differences between groups. Download FIG S3, TIF file, 0.1 MB. Copyright © 2017 Sugi et al. 2017 Sugi et al. This content is distributed under the terms of the Creative Commons Attribution 4.0 International license.

### A phosphomimetic TgGP^S25E^ mutant could compensate an amylopectin accumulation phenotype due to knockout of TgCDPK2.

**FIG 4:**
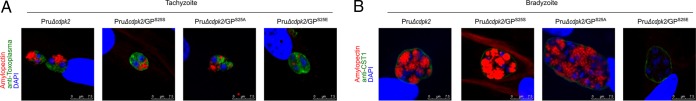
GP^S25E^-3myc compensated for amylopectin accumulation caused by knockout of CDPK2. CDPK2 knockout parasites with mutated GP-3myc expression were inoculated to HFF host cells and incubated for 24 h under normal culture conditions for tachyzoites (A) or incubated for 2 h to allow the parasites to invade host cells, followed by incubation under conditions of pH 8.2 medium without CO_2_ for 4 days for bradyzoites (B). Parasites were stained with PAS (red), followed by immunofluorescence detection of parasites (green) (anti-*Toxoplasma* rabbit polyclonal antibody for tachyzoites or anti-CST1 monoclonal antibody for bradyzoites). Nuclei were stained with DAPI (blue). Scale bars show 7.5 µm.

To determine if the Pru/GP^S25E^ strain had hyperactive amylopectin digestion, we introduced a CDPK2 knockout into this strain. Knockout of CDPK2 has previously been demonstrated to cause amylopectin accumulation (15). Consistent with this previous report, our CDPK2 knockout parasites, with a stop codon mutation in the first exon before the kinase catalytic domain, demonstrated amylopectin accumulation under both tachyzoite and bradyzoite culture conditions in parasites with a wild-type TgGP (Pru*Δcdpk2*) or in parasites with a silent mutation at S25 (Pru*Δcdpk2/*GP^S25S^) (Fig. 4A and B; Fig. S2B). CDPK2 knockout parasites that had inactive GP (Pru*Δcdpk2*/GP^S25A^) also had amylopectin accumulation as expected (Fig. 4A and B; Fig. S2B). In contrast, the Pru*Δcdpk2*/GP^S25E^ strain displayed almost no amylopectin accumulation in the tachyzoite stage (Fig. 4A) and no amylopectin was detected in bradyzoites (Fig. 4B). Quantitative analysis of amylopectin using PAS staining confirmed an absence of amylopectin (*P* < 0.001) in the Pru*Δcdpk2*/GP^S25E^ strain (Fig. S2B). These data clearly suggest that GP^S25E^ is hyperactive, leading to amylopectin digestion, and that the amylopectin accumulation caused by CDPK2 knockout was compensated by the digestive effect of GP^S25E^.

### Second-copy expression of active TgGP restored normal amylopectin digestion in the Pru/GPS^S25A^ strain.

**FIG 5:**
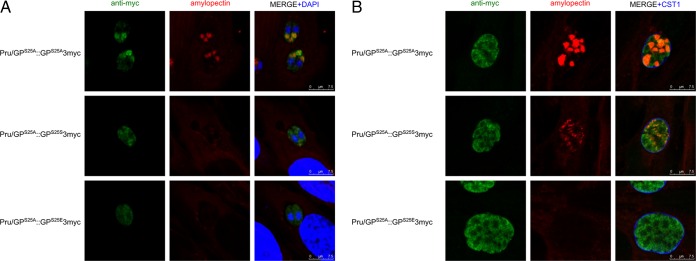
Expression of active TgGP-3myc variants in Pru/GP^S25A^ restored amylopectin digestion. Pru/GP^S25A^ parasites were transfected with plasmids expressing TgGP-3myc with various mutations of GP Ser25 as indicated in the figure. Parasites were inoculated to HFF host cells and incubated for 24 h under normal culture conditions for tachyzoites (A) or incubated for 2 h to allow the parasites to invade host cells, followed by incubation under conditions of pH 8.2 medium without CO_2_ for 4 days for bradyzoites (B). Cells were fixed and stained to detect amylopectin and the TgGP-3myc mutant. It should be noted that after the PAS-staining procedure, a GFP signal could no longer be observed. TgGP1-3myc (green), PAS-positive amylopectin (red), and nuclei (blue) are shown. Scale bars show 7.5 µm.

We expressed mutated GP-3myc as a second copy in Pru/GP^S25A^ to identify the location of the mutated TgGP in the parasite and to verify whether the amylopectin digestion defect in Pru/GP^S25A^ was caused by the Ser25 mutation. The expression of GP^S25A^-3myc in Pru/GP^S25A^ did not restore amylopectin accumulation in either tachyzoites (Fig. 5A) or bradyzoites (Fig. 5B). GP^S25A^-3myc was found to colocalize to the site of PAS-positive amylopectin signals, suggesting that inactive GP^S25A^ was still targeted to its amylopectin substrate even if the enzyme was no longer functional for digestion. The expression of GP^S25S^-3myc or GP^S25E^-3myc restored to normal (e.g., to wild type) the amylopectin digestion defect seen in Pru/GP^S25A^ (Fig. 5). Both GP^S25S^-3myc and GP^S25E^-3myc localized to the cytosol of the parasite in a patched fashion (Fig. 5) that was similar to the GP-3myc localization seen in wild-type GP (Fig. 1). GP^S25S^-3myc colocalized to amylopectin sites in bradyzoites and was also seen in the cytosol in a patched manner (Fig. 5B). These data indicate that the GP^S25A^ mutation caused an amylopectin digestion defect in *T. gondii* and that both GP^S25S^ and GP^S25E^ were active enzymatically and able to restore wild-type amylopectin digestion. Because GP^S25E^ has already been identified as a hyperactive GP causing a defect in amylopectin storage in bradyzoite stages (Fig. 3B and 4), we further used the Pru/GP^S25A^::GP^S25S^-3myc complemented parasite and demonstrated that the introduction of GP^S25A^::GP^S25S^-3myc restored the proper regulation of amylopectin digestion.

### Altered amylopectin digestion in *T. gondii* resulted in reduced cyst numbers in parasite-infected mouse brains.

**FIG 6:**
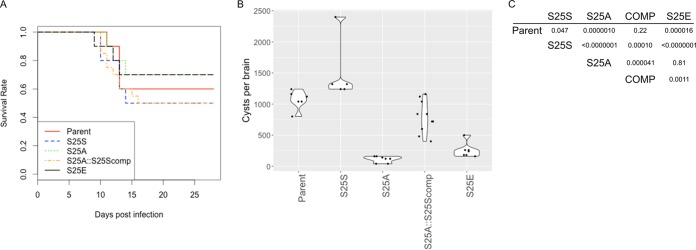
Regulation by Ser25 of TgGP is required for the efficient production of the brain cyst. One thousand freshly isolated tachyzoites were injected intraperitoneally into 6- to 8-week-old C57BL/6J female mice. (A) Survival curves of the infected mice. For 4 weeks after infection, dead mice were recorded daily. Statistical tests were performed to detect differences among the groups by Gehan-Wilcox test (*P* = 0.899). Ten mice each were used for the parental *T. gondii* strain and the Pru/GP^S25S^, Pru/GP^S25A^, and Pru/GP^S25E^ mutants, and 20 mice were used for *T. gondii* Pru/GP^S25A^∷GP^S25S^-3myc (complement). (B) At 4 weeks postinfection, tissue cysts in the brains of surviving mice were counted. The cyst numbers per brain are shown as a violin plot for each group, with each single data point showing the result for an individual mouse. One-way ANOVA and Tukey’s *post hoc* test were used to detect statistical differences between mean cyst number values of each group. (C) The adjusted *P* value for each pairwise comparison by Tukey’s HSD test is shown.

Finally, to investigate a role in the regulation of amylopectin digestion in the biological context, we used the various GP mutants to infect mice and examined cyst production in these infected animals. Parental strain Pru*Δku80Δhxgprt* and mutant strains Pru/GP^S25S^, Pru/GP^S25A^ (mutant parasite that lacks amylopectin digestion and was packed with amylopectin in the bradyzoite), and Pru/GP^S25E^ (mutant parasite that has a hyper-amylopectin digestion that resulted in the lack of amylopectin even in the bradyzoite stage) were inoculated into C57BL/6J mice. In the acute phase of the infection, there were no significant changes in the mortality rates of infected mice among the groups (Fig. 6A). At the latent infection stage, 4 weeks postinfection, brains were collected and the cyst number in each brain was determined. Pru/GP^S25A^ and Pru/GP^S25E^ demonstrated reductions in cyst numbers compared to the cyst numbers of the parental Pru*Δku80Δhxgprt* and Pru/GP^S25S^ *T. gondii* strains (Fig. 6B and C). The reduced cyst number of the Pru/GP^S25A^ strain was restored in complemented parasites, e.g., Pru/GP1^S25A^::GP1^S25E^-3myc (Fig. 6B and C). All of the mutant parasites utilized for mouse infections formed plaques of similar sizes under tachyzoite culture conditions, indicating that *in vitro* parasite growth was not altered significantly by the various mutations introduced into GP Ser25 (Fig. S4). These data suggest that not only excess amylopectin accumulation but, also, a lack of amylopectin accumulation resulted in reduced cyst numbers and support our underlying hypothesis that proper control of amylopectin digestion in *T. gondii*, which is achieved by regulation at the GP Ser25 site, is required for the development of an effective persistent infection.

10.1128/mBio.01289-17.4 FIG S4  Pru/GP mutants did not have significant growth defects in the *in vitro* plaque assay. The parasite lines used for the *in vivo* infection study were examined for growth rates using a published plaque assay (14, 30). Representative images of plaques in 6-well plates infected with 100 parasites followed by 10 days of culture are shown. Plaque sizes were normalized to the mean plaque size of the parental parasite. Violin plots with individual dots indicating plaques are shown. One-way ANOVA analysis did not demonstrate any significant differences between mean values of the groups. Download FIG S4, TIF file, 1 MB. Copyright © 2017 Sugi et al. 2017 Sugi et al. This content is distributed under the terms of the Creative Commons Attribution 4.0 International license.

## DISCUSSION

Polysaccharide granule storage can be visualized by ultrastructural studies of *T. gondii* (4), and these visualized granules have been biochemically characterized as containing glucose polymers in the form of plant-type amylopectin (5, 6). The same amylopectin molecules are found in related organisms, such as *Eimeria* and *Cryptosporidium*. Nakai and Ogimoto reported that the amount of stored amylopectin in *Eimeria* sporozoites has a direct relation with sporozoite infectivity (8), and Jenkins et al. reported that *Cryptosporidium* sporozoite viability in the oocyst also is related to the amount of amylopectin it contains (7). These data suggest that the energy storage function of amylopectin may be critical for the nutrient-poor environment found outside host cells. In *Toxoplasma gondii*, amylopectin storage is known to increase in bradyzoites, the latent-infection life cycle stage, as well as in sporozoites, macrogametocytes, and oocysts (sexual reproductive stages found in the intestine of cats, the definitive host), whereas amylopectin is not found in merozoites or in microgametocytes (sexual reproductive stages found in the cat intestine) and only a few granules are present in tachyzoites (reviewed in reference 4). The function and regulatory mechanisms of this storage system, especially in bradyzoites, are not fully elucidated. Herein we identified that either an amylopectin digestion defect or an amylopectin hyperdigestion state (e.g., the absence of amylopectin storage), altered murine brain cyst burdens.

Recently, Uboldi et al. (15) reported that *Δcdpk2* parasites with amylopectin accumulation no longer made tissue cysts in the infected mice. Because *Δcdpk2* parasites are packed with amylopectin and have an aberrant morphology (15), the possibility that the lack of detectable cysts in infected mouse brains resulted from morphological destruction could not be excluded. In this report, we used parasites expressing either an inactive GP (packed with amylopectin) or a hyperactive GP (lacking amylopectin) and demonstrated that the proper regulation of amylopectin metabolism is required for persistent infection. Moreover, we determined that the Ser25 site of GP is a critical residue in the regulation of the balance between amylopectin storage and digestion.

Genome-wide screening for gene fitness suggests that disruption of TgGP (in the type I RH strain) does not affect fitness under tachyzoite culture conditions (phenotype enrichment score of 1.01) (16). A direct competition assay with parasites with a silence mutation or stop codon mutation at the first exon of TgGP has shown that around 2 to 3 weeks are required to detect a growth difference between the TgGP knockout strain and wild-type parasites in a Pru*Δku80Δhxgprt* strain background (17). This suggests that TgGP is dispensable for tachyzoite growth under normal tissue culture conditions.

Phosphorylation of the N-terminal end of the glycogen phosphorylase is well documented for mammalian-type glycogen phosphorylase. We identified that GP Ser25 is a critical residue for the proper regulation of amylopectin digestion in *T. gondii*; however, the direct upstream protein kinase remains unknown. The regulatory Ser phosphorylation of mammalian and yeast GP is provided primarily by phosphorylase kinase PhK (18) and partially by cAMP-dependent protein kinase PKA (12). An ortholog of PhK is, however, not present in the *Toxoplasma* genome (http://www.ToxoDB.org). One of the candidate protein kinases that could contribute to regulation of TgGP is TgCDPK2, whose knockout has been reported to result in amylopectin accumulation (15). The phosphomimetic mutant GP^S25E^ was demonstrated to complement the amylopectin accumulation phenotype seen in *Δcdpk2* strains. However, this reversion of amylopectin accumulation does not prove that Ser25 phosphorylation is due to CDPK2. Uboldi et al. did not observe any significant difference in the amount and presence of the Ser25-phosphorylated GP peptide compared to the total peptides found for GP in a proteomic analysis of the amylopectin-bound soluble fraction isolated from *Δcdpk2* parasites (15). These data suggest that the S25E mutation might compensate the amylopectin accumulation through a CDPK2-independent pathway. A quantitative analysis of the phosphoproteome in a CDPK2 knockout mutant will be required to conclude whether CDPK2 can or cannot directly regulate TgGP Ser25.

The result that Pru*Δcdpk2*/GP^S25S^ had more amylopectin accumulation than Pru*Δcdpk2*/GP^S25E^ suggests that not all of the GP Ser25 is phosphorylated in either the tachyzoite or bradyzoite stage in the *Δcdpk2* parasite. CDPK2 has been shown to catalyze the phosphorylation of PPDK, and the *Δcdpk2* parasite has been shown to have reduced phosphorylation on water dikinase (WDK) (15); this suggests that a regulatory function of CDPK2 is to regulate the phosphorylation mark on amylopectin to initiate the partial digestion of amylopectin, which is upstream from GP (Fig. 1A) (19). GP can catalyze alpha 1-4-linked glucose; however, full degradation of the intact starch molecule requires phosphoglucan phosphatase (19), which removes the inhibitory phosphorylation marks on the starch made by WDK and PPDK (Fig. 1A) (20), and the debranching enzyme of 1-6-linked starch branches (EC 2.4.1.25); otherwise, GP catalysis will stop at the fourth glucose unit, before the 1-6-linked branching sites (12). Our results, however, demonstrating successful complementation of the *Δcdpk2* amylopectin phenotype with GP^S25E^, suggest that CDPK2 does not just affect the upstream digestion pathway of debranching and phosphorylation marks on amylopectin. Another possibility is that hyperactive GP^S25E^ digests the glucose polymer before the parasite stores the branched and phosphorylation-marked starch. A detailed analysis of each phosphorylation site of the enzymes in the amylopectin metabolism pathway and the functional effects of these posttranslational modifications will be required to fully understand the underlying regulatory mechanisms of this critical energy storage system.

Another possible upstream kinase of GP is PKA. GP Ser25 sits in the sequence motif [R/K][R/K]XS, which is known to be the typical substrate of the cAMP-dependent protein kinase PKA (21). *T. gondii* has three distinct PKA catalytic subunits (TgPKAc1, -2, and -3) (14). Of these three PKA subunits, TgPKAc3 has been shown to have a role in bradyzoite regulation/differentiation, and knockout of TgPKAc3 results in a bradyzoite-like phenotype (14). Analysis of the *Δpkac3* strain shows that it does not display a residual body packed with amylopectin, which is visible both in the GP mutants and in the CDPK2 knockout strain; therefore, we do not believe that TgPKAc3 is the kinase involved in CP Ser25 phosphorylation. It is possible, however, that S25 phosphorylation could be compensated by other protein kinases, such as TgPKAc1 and CDPK2 in the *Δpkac3* strain.

The amylopectin accumulation phenotype is a consequence of the alteration of the balance of synthesis and digestion, which is also controlled by transcriptional regulation of the related enzymes. Coppin et al. have shown using reverse transcription (RT)-PCR that glycogen synthase is more expressed in the tachyzoite and TgGP is less expressed in the bradyzoite stage (6), suggesting that tachyzoites have more potential for amylopectin accumulation if the regulatory posttranslational modification is not considered. *T. gondii Δpkac3* has a bradyzoite-like phenotype (14), and this could affect downregulation of amylopectin synthesis enzymes at the transcription level. A further analysis of quantitative peptide phosphorylation of TgGP Ser25 mutant parasites in which various candidate protein kinases (e.g., PKAc3 or CDPK2) have been ablated would likely demonstrate a role of PhK-independent kinase signals in starch metabolism, which has been described in other eukaryotic organisms (12).

Phosphomimetic activity due to Glu produces a negative charge at the GP Ser25 position. However, phosphorylated Ser has two negative charges, whereas Glu has only a single negative charge. The crystal structure of phosphorylated mammalian GP has shown that the N-terminally phosphorylated Ser makes two electrostatic bonds with the counterfacing positively charged amino acids in a homodimeric formation (between S14^phos^ and R43 of its own subunit and the R69 from the other dimer subunit) (12). Interestingly sequence alignment of the GP N-terminal domain shows that one of the positively charged amino acids (equivalent to R69) is not conserved in the apicomplexan GPs (Fig. S1 in the supplemental material). This partial loss of the interacting amino acid site may explain why the Glu-for-Ser mutation resulted in a highly active TgGP in this parasite.

While *T. gondii Δcdpk2* did not form brain cysts (15), our inactive-GP S25A mutant, which had a similar amylopectin accumulation phenotype, produced brain cysts. This difference could partially reflect the detection levels of brain cysts, because our control strain showed 500 to 1,000 cysts per brain, which is similar to the numbers in previous reports (22) and almost 10 times more than for the wild-type control in the paper on *Δcdpk2* (15). Alternatively, this could reflect different mechanisms involved in amylopectin accumulation that resulted in different degrees of the *in vivo* cyst formation phenotype. For example, our GP mutants only displayed changes in their GP enzymatic activity, whereas *Δcdpk2* mutants showed changes in both synthesis and digestion of amylopectin, as well as alterations in multiple targets due to the loss of kinase activity (15). Because one can obtain brain cysts with changes in amylopectin by using the GP mutants we have developed, these GP mutants will allow studies on the effects of changes in amylopectin content on cyst functions, such as the infectivity of these cysts for mice and for cats, the ability of cysts to result in the development of feline sexual life cycle stages, and the length of survival of these cysts.

The reduction in cyst numbers seen with the hyperactive-GP mutant, which did not have amylopectin accumulation, indicates that proper regulation of digestion itself affected the parasite’s biology and the cyst number. Bradyzoites in mature tissue cysts in mouse brains have been demonstrated to be a nonreplicating quiescent stage (G0) rather than an actively replicating stage (23). Time-dependent kinetics of cyst numbers in infected mouse brains has demonstrated that replication does occur in cysts early in infection, as cyst numbers increase between 2 and 3 weeks following primary infection (24). Live imaging of the *in vitro* bradyzoite has shown that a parasite vacuole can have fission to produce a new adjacent tissue cyst without rupturing its host cell (25). These kinds of switching from nonreplicating parasites to replicating parasites or forming a new cyst during the persistent infection stage probably require the utilization of amylopectin as an energy storage source. Further investigations of the changes in the energy demand of bradyzoites during their host’s life cycle and the underlying biological events experienced by bradyzoites will extend our understanding of latency and persistent infection due to *Toxoplasma gondii*.

### Conclusions.

In the complex stage transitions of this parasite, amylopectin energy metabolism is hypothesized to serve as an energy reservoir to adapt to environmental changes. By use of the mutants with inactive and hyperactive GP, we revealed that a balance of storage and digestion of amylopectin is critical for *T. gondii*’s development and latency. Because amylopectin is also observed in the cat intestine stage and oocyst/sporozoite stages, further investigation of the role of amylopectin metabolism in the function of these life cycle stages is also needed. Studies of the role of amylopectin storage should result in an improved understanding of the mechanisms of environmental adaptation of this parasite. Manipulation of GP activity in the parasite, by the amino acid substitutions at Ser25, provides an important tool for investigating the role of amylopectin in multiple *T. gondii* life cycle stages.

## MATERIALS AND METHODS

### Cell culture.

Human foreskin fibroblasts (HFF) were obtained from ATCC and maintained in Dulbecco’s modified Eagle’s medium (DMEM) (Life Technologies, Inc.) supplemented with 10% fetal bovine serum (FBS), penicillin, and streptomycin (Life Technologies, Inc.). Pru*Δku80Δhxgprt* (22) parasites and mutant parasites derived from this parental parasite strain were maintained in confluent HFF host cells using the same culture medium.

### Sequence analysis.

For the phylogenetic analysis of the GP genes, alpha-glucan phosphorylase protein sequences were retrieved from Uniprot (http://www.uniprot.org/) and a multiple alignment was performed using ClustalW (Fig. S1). A phylogenetic relation among GPs was inferred by using the maximum-likelihood method based on the Le_Gascuel_2008 model (26). The tree with the highest log likelihood (−10955.1618) is shown. The percentage of trees in which the associated taxa clustered together is shown next to each branch. Initial tree(s) for the heuristic search were obtained by applying the neighbor-joining method to a matrix of pairwise distances estimated using a JTT model. A discrete Gamma distribution was used to model evolutionary rate differences among sites (5 categories [+G, parameter = 1.5903]). The tree is drawn to scale, with branch lengths measured in the number of substitutions per site (Fig. 1B). The analysis involved 10 amino acid sequences. There were a total of 1,098 positions in the final data set. Evolutionary analyses were conducted in MEGA6 (27).

### Transgenic parasites.

The transgenic parasites used in this report are listed in Table 1. To express the second copy of TgGP with a C-terminal 3myc tag, the genomic region of TgGP (TGME49_310670) including the 5′ untranslated region (UTR) was PCR amplified with primers GP-express-1 and GP-express-2 (Table S1 in the supplemental material) and cloned into pLIC-3myc-HXGPRT using Gibson assembly (NEB) according to the manufacturer’s instructions.

10.1128/mBio.01289-17.5 TABLE S1  Primers utilized for the studies described. Download TABLE S1, PDF file, 0.1 MB. Copyright © 2017 Sugi et al. 2017 Sugi et al. This content is distributed under the terms of the Creative Commons Attribution 4.0 International license.

To mutate a codon at the Ser25 position of GP in the native genome locus, p-DHFR-Cas9GFP-sgGP (single guide RNA [sgRNA] target sequence, GCTTGGAAAATGACGCCTTT) and donor DNA were prepared as previously described (17) using primers listed in Table S1. After cotransfection of the circular p-DHFR-Cas9GFP-sgGP and linearized donor DNA as described in reference 17, parasites which incorporated transfected DNA were selected using 1 µM pyrimethamine for 10 days. After the initial selection, the drug was removed, parasites were cloned by limiting dilution, and the genotype was checked by sequencing the target region. To complement Pru/GP^S25A^ parasites with second-copy expression of various TgGP mutants, the genomic region of TgGP in the mutated parasites was PCR amplified with the same primers as used in the step described above and cloned into pLIC-3myc-HXGPRT.

To introduce a stop codon into the first exon of CDPK2, pHXGPRT-Cas9GFP-sgCDPK2 and donor DNA were prepared with primers listed in Table S1. Parasites were cotransfected with 5 µg of circular pHXGPRT-Cas9GFP-sgCDPK2#2 and 1.6 µg of the double-stranded DNA (dsDNA) donor with a stop codon mutation. After transfection, parasites were selected with 25 µg/ml mycophenolic acid and 50 µg/ml xanthine for 10 days to enrich the parasite population that incorporated transfected DNA at the time of transfection. After limiting dilution, mutant parasites were screened with PCR-restriction fragment length polymorphism (RFLP) of the target region and checked for the stop codon mutation by Sanger sequencing. It should be noted that for CDPK2 mutagenesis, we picked three sgRNA candidates and checked them for their cleavage efficiency before determining the target codon, as described in reference 28. Briefly, strain RH*Δhxgprt* was transfected with 5 µg pHXGPRT-Cas9GFP-sgCDPK2#1, -2, or -3 and selected with mycophenolic acid and xanthine to enrich the transfected parasites. After enrichment, these parasites were used for gDNA extraction. The target region was PCR amplified and sequenced to detect the mixed chromatograms after the cleaved site. Only with sgCDPK2#2 did we observe evidence of cleavage, and we used this construct for introducing stop codon mutations into Pru*Δku80Δhxgprt*-derived mutants.

### Bradyzoite induction.

Bradyzoite induction was conducted as described in reference 14. Briefly, parasites were inoculated to confluent HFF monolayers cultured on glass coverslips. Two hours after the inoculation, the culture medium was replaced with DMEM, pH 8.2, without sodium bicarbonate and with 50 mM HEPES, supplemented with 1% FBS and with penicillin and streptomycin (bradyzoite-inducing medium). The infected cells were cultured without added CO_2_ with medium changes every 2 days for 4 days.

### Immunostaining-compatible PAS staining.

To visualize the amylopectin in the parasite with other parasite antigen markers, we used a modified periodic acid-Schiff (PAS) staining technique that is compatible with immunostaining (29). Prior to staining, parasites were inoculated to host monolayers cultured on the coverslips and cultured for 24 h for the tachyzoite conditions or for 4 days under bradyzoite induction conditions as described above.

### Fixation and permeabilization for PAS staining.

Infected cells were fixed with 4% paraformaldehyde in phosphate-buffered saline (PBS) for 30 min at room temperature and washed with PBS. Excess fixatives were quenched, and cell membranes were permeabilized in 0.2% Triton X-100 and 0.2 M glycine in PBS for 20 min at room temperature. Cells were washed with PBS three times and rinsed with distilled water once before the PAS-staining steps.

### Modified PAS staining followed by immunostaining.

Cells were reacted with 1% periodic acid (Sigma) for 5 min, washed for 1 min with tap water, and rinsed once with distilled water, followed by incubation with Schiff’s reagent (Sigma) for 15 min at room temperature. After the reaction steps, the samples were rinsed with distilled water and washed in tap water for 10 min to develop PAS staining. After this washing, the samples were washed three times with PBS, blocked with 3% bovine serum albumin (BSA) and 0.1% Tween 20 in PBS (PBS-T) (blocking buffer), and incubated with anti-cMYC rabbit monoclonal antibody (Cell Signaling Technology), anti-*Toxoplasma* rabbit antisera (14), anti-hemagglutinin (HA) rat monoclonal antibody clone 3F10 (Sigma), or anti-CST1 monoclonal antibody (clone SalmonE) (30) (500:1 in blocking buffer at 4°C overnight). After the primary antibody staining, samples were washed with PBS-T and incubated with Alexa 488-conjugated secondary antibodies for rabbit or rat, CF405-conjugated anti-mouse secondary antibody (1:1,000 in blocking buffer, room temperature for 1 h), and 4′,6-diamidino-2-phenylindole (DAPI) for nuclear staining. Stained samples were washed with PBS-T and mounted with ProLong gold mounting medium (Thermo Fisher). It should be noted that the green fluorescent protein (GFP) signal that we could observe within bradyzoites of Pru*Δku80Δhxgprt* was diminished after the PAS-staining steps. Images were collected with a Leica TCS SP5 confocal microscope. The PAS-positive signal was detected by the red fluorescence excited with a 543-nm laser line.

### Amylopectin quantification.

*In vitro* bradyzoites were stained with a 1:6 dilution of Schiff’s reagent in distilled water following fixation, permeabilization, and periodic acid treatment as described above. Cysts were identified by staining with anti-CST1 monoclonal antibody (clone salmonE [30]) followed by incubation with Alexa 488-conjugated anti-mouse secondary antibody (1:500 in blocking buffer at 37°C for 1 h). PAS-positive signals were detected using a G1B Nikon fluorescence filter cube, while CST1-positive signals were detected using a B2A Nikon fluorescence filter cube. Images of cysts were obtained on a Nikon Diaphot 300 inverted epifluorescence microscope (Nikon Instruments, Inc.) using 2-s exposures on a Tucsen H series digital camera (Fuzhou Tucsen Photonics Co., Ltd.). The PAS signal contained within cysts was measured in ImageJ (https://imagej.nih.gov/ij/) by obtaining the mean fluorescence intensity. The fluorescence intensity data produced by ImageJ was processed and visualized using the statistical program R (https://www.r-project.org/), and the significance of differences among the various mutant strains in relation to amylopectin staining (PAS signal) was calculated using the Kruskal-Wallis test followed by Dunn’s test with a Bonferroni-Holm adjustment for *post hoc* comparisons.

### Murine *in vivo* infection and cyst enumeration.

Ten female 6- to 7-week-old C57BL/6J mice (The Jackson Laboratory, Bar Harbor, ME) per group were infected with 1,000 purified tachyzoites intraperitoneally. For the mouse experiments with complemented strain Pru/GP1^S25A^::GP1^S25S^-3myc, 20 mice were used. To determine the brain cyst burdens, at 4 weeks postinfection, surviving mice in each group were euthanized and brains were collected to count the brain cysts. The brains were cut in half, and one half hemisphere was fixed in 4% paraformaldehyde in PBS overnight at 4°C and homogenized in 10 ml PBS. The homogenized cells were centrifuged at 1,000 × *g* for 5 min, and the pellet was resuspended in 1 ml PBS. Fifty microliters of the homogenate was inspected under a fluorescence microscope to count the GFP-positive cyst number to estimate the cyst number per brain.

### Plaque assay.

Purified tachyzoites of each mutant parasite were inoculated into a 6-well culture plate containing a monolayer of host HFF cells. Twenty, 100, and 500 parasites per well were used for infection for each group. After 10 days of culture without disturbance, cells were washed with PBS and fixed with 4% paraformaldehyde in PBS for 20 min at room temperature. After fixation, immunostaining was conducted to visualize the edge of the lysed host cell area (plaque). Briefly, the fixative was quenched and fixed cells were permeabilized with 0.2-M glycine and 0.2% Triton X-100 in PBS for 20 min. Cells were blocked with blocking buffer (1% BSA in 0.1% Tween 20–Tris-buffered saline [TBS-T]) for 30 min at 37°C and then incubated with anti-*Toxoplasma* rabbit polyclonal antisera in blocking buffer (dilution of 1:4,000) at 37°C for 1 h, followed by three TBS-T washes. Cells were then incubated with alkaline phosphatase-conjugated anti-rabbit secondary antibody (Millipore) in blocking buffer (dilution of 1:5,000) for 1 h at 37°C, followed by three TBS-T washes. Immunostaining signals were developed by using BCIP-NBT (5-bromo-4-chloro-3-indolylphosphate–nitroblue tetrazolium) substrate (G-Biosciences and Thermo Fisher) for 10 min at room temperature with shaking, followed by three rinses with distilled water. Stained plates were scanned with a document scanner, and images were analyzed with ImageJ to measure the size of plaques.

### Statistics.

To compare the multiple groups, one-way analysis of variance (ANOVA) followed by the *post hoc* Tukey honestly significant difference (HSD) test was employed. To compare the survival curves for mice infected with mutated parasites, the Gehan-Wilcox test was performed using the R package “survival” (https://cran.r-project.org/web/packages/survival/index.html) (31).

### Ethics statement.

All animal experiments were conducted according to the United States Public Health Service Policy on Humane Care and Use of Laboratory Animals. Animals were maintained in an AAALAC-approved facility, and all protocols were approved by the Institutional Care Committee of the Albert Einstein College of Medicine, Bronx, NY (animal protocols 20150908, 20121104, 20121109, and 20121110 and animal welfare assurance no. A3312-01). No human samples were used in these experiments. Human foreskin fibroblasts were obtained from the ATCC.
