# Supplementary figures and images for: Toxoplasma gondii Requires Glycogen Phosphorylase for Balancing Amylopectin Storage and for Efficient Production of Brain Cysts
Source: mBio. 2017 Aug 29;8(4):e01289-17. doi: 10.1128/mBio.01289-17 (PMC5574715; doi:10.1128/mBio.01289-17)

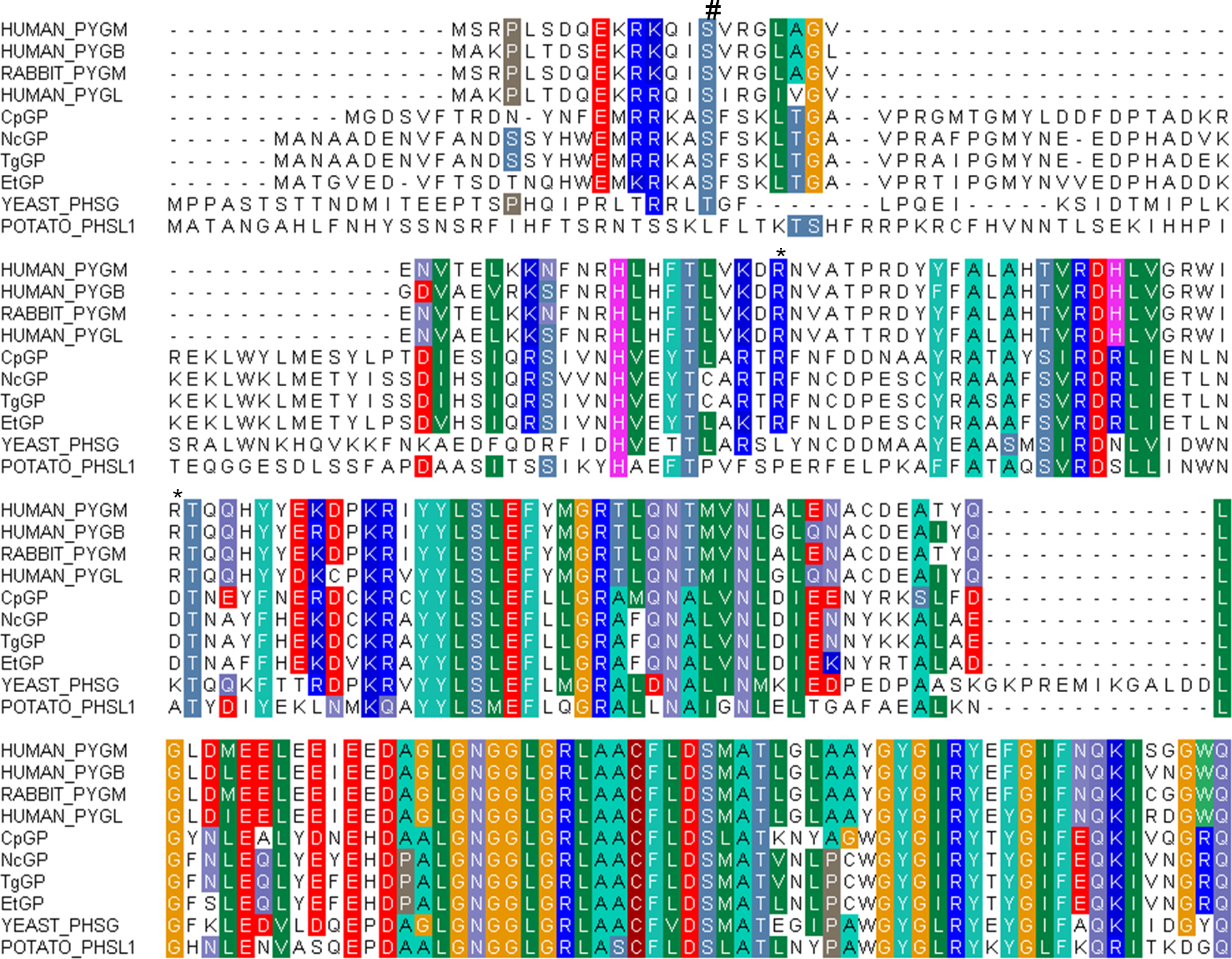

Supplement: FIG S1 [file mbo004173451sf1.tif]

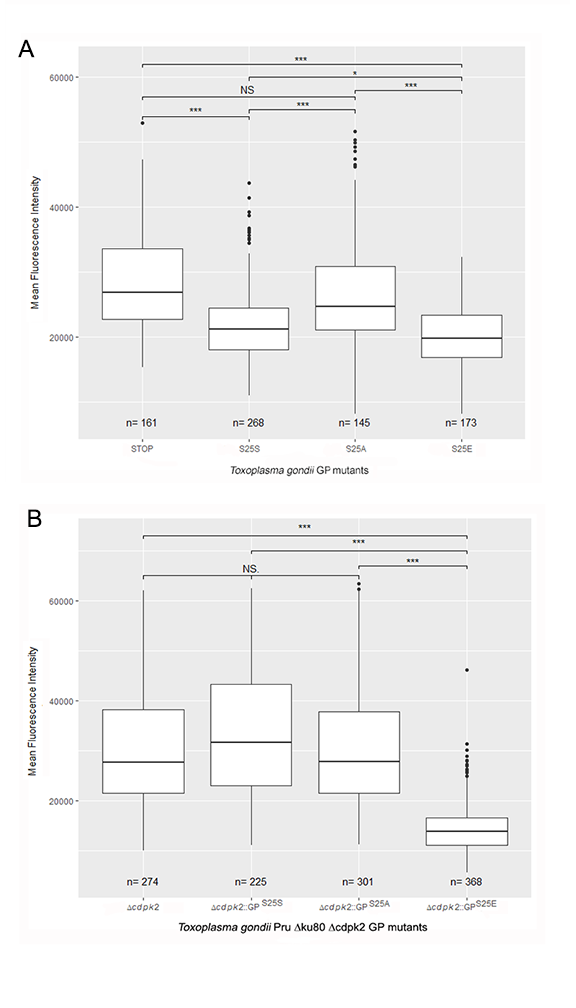

Supplement: FIG S2 [file mbo004173451sf2.tif]

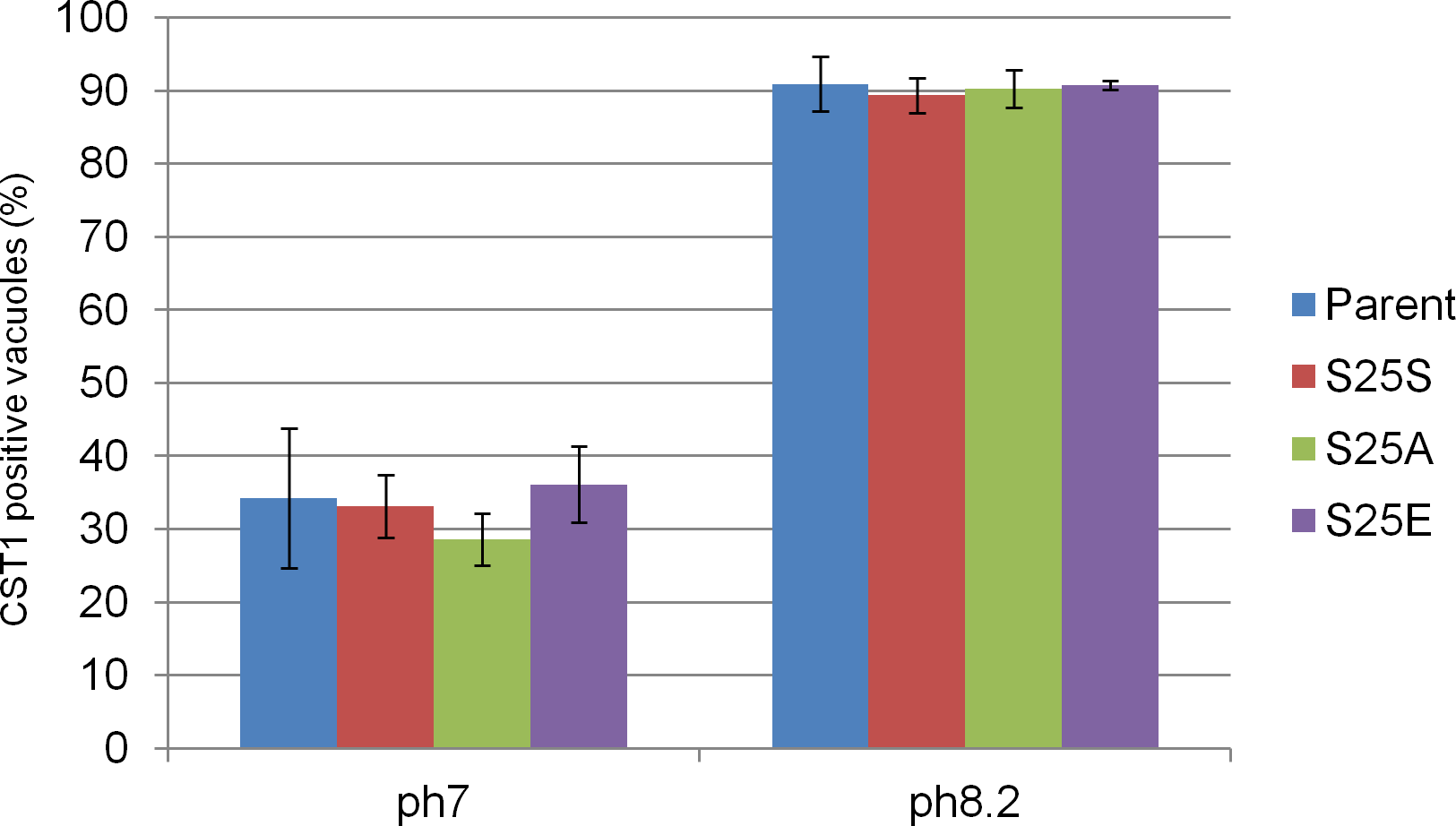

Supplement: FIG S3 [file mbo004173451sf3.tif]

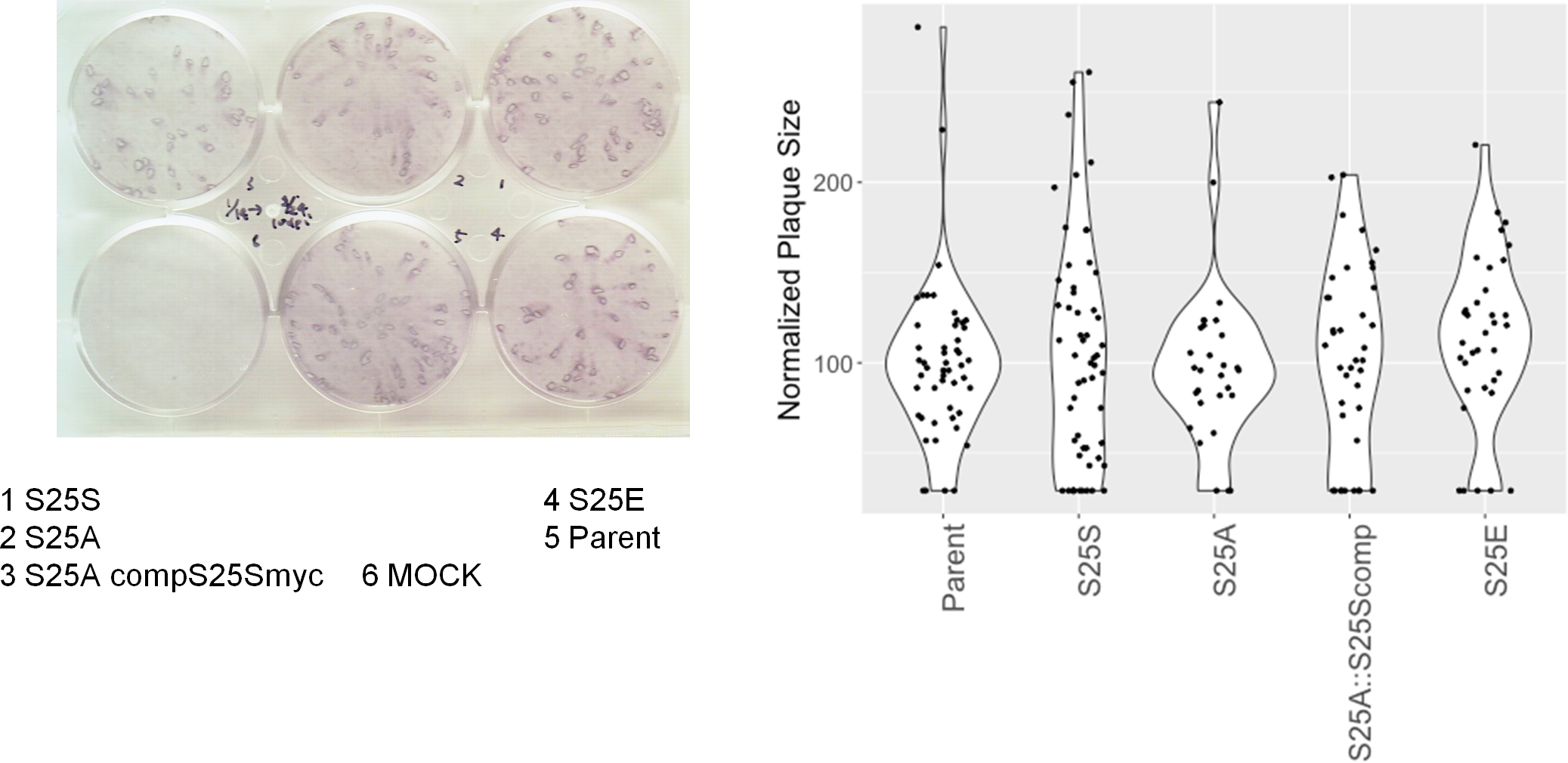

Supplement: FIG S4 [file mbo004173451sf4.tif]
