# Supplementary material for: Toxoplasma gondii Requires Glycogen Phosphorylase for Balancing Amylopectin Storage and for Efficient Production of Brain Cysts
Source: mBio. 2017 Aug 29;8(4):e01289-17. doi: 10.1128/mBio.01289-17 (PMC5574715; doi:10.1128/mBio.01289-17)
Supplement: TABLE S1 [file mbo004173451st1.pdf]

Supplemental Table 1. Primers used in the present study

name	sequence 5'-->3'	usage
GP-express-1	TGATTACGCCAAGCTCGGAAGCAGTTCTGAATCTCTGGTG	ampification of TgGP genomic region for expression contruct
GP-express-2	GTCCTCCACTTCCAATCGCTGAGACTTTGTTTGGGATG	ampification of TgGP genomic region for expression contruct
pLIC_MYC_F	ATTGGAAGTGGAGGACGGGAA	linearization of pLIC-3MYC-HXGPRT
pLIC_R	TTCCGAGCTTGGCGTAATCA	linearization of pLIC-3MYC-HXGPRT
sgRNA-R	AACTTGACATCCCCATTTAC	ref(a), making cas9-dhfr-sgRNA with forward primers
GP-sgRNA-F	GCTTGGAAAATGACGCCTTTGTTTTAGAGCTAGAAATAGC	ref(a), making cas9-dhfr-sgRNA for GP
GP1_donor_F	TATCTCATCCGGGACAGCGT	ref(a), making donor seq for GP
GP1_donor_R	CGAGTTCTAGCGCAGGTGTA	ref(a), making donor seq for GP
donor_mut_R	CGCCTTTCGtCGCATTTCCCAATGGTACGAC	ref(a), making mutation in donor seq
donor_mut_S_F	tccTTTTCCAAGCTGACCGGAGCAGTG	ref(a), making silent mutation in donor seq
donor_mut_stop_F	taaTTTTCCAAGCTGACCGGAGCAGTG	ref(a), making stop codon mutation in donor seq
donor_mut_A_F	gcaTTTTCCAAGCTGACCGGAGCAGTG	making S25A mutation in donor seq
donor_mut_E_F	gaaTTTTCCAAGCTGACCGGAGCAGTG	making S25E mutation in donor seq
GP-trackR	CGCGTTCGTGTCGTTAAGTG	amplification of mutated locus with GP-donor-F
CDPK2-sgRNAF1	CTTTCCAGGTGACTTGCTCAGTTTTAGAGCTAGAAATAGC	making cas9-hxgprt-sgCDPK2-1
CDPK2-sgRNAF2	CATGAGCAAGTCACCTGGAAGTTTTAGAGCTAGAAATAGC	making cas9-hxgprt-sgCDPK2-2
CDPK2-sgRNAF3	GAAAGAACCACCGAGTTCTGGTTTTAGAGCTAGAAATAGC	making cas9-hxgprt-sgCDPK2-3
CDPK2-donorStopTemplate	AGAGATGCCGCTCAAGACTTCCTGGCATTGTTCGTGCAACGCaACgTTTtgAGGTGACTTGCTCATGGTTGTTGCTAACCACGATCGCGTGGGCAACTGG	Making stop codon mutation donor for CDPK2 (template)
CDPK2-donorRevPrimer	AGAACCACCGAGTTCTGAGGATTCCAGTTGCCCACGCGAT	Making stop codon mutation donor for CDPK2
CDPK2-screen-F	CTGCGGAATTTGTGCGTGAG	amplification of CDPK2 target locus, and sequence
CDPK2-screen-R	AGGACGTATTCGCAGTCAGC	amplification of CDPK2 target locus

(a) Sugi T, Kato K, Weiss LM. An improved method for introducing site-directed point mutation into the Toxoplasma gondii genome using CRISPR/Cas9. Parasitol Int. Elsevier Ireland Ltd; 2016; 1–5. doi:10.1016/j.parint.2016.05.002
